# Case Report: Genetic and immune microenvironmental characteristics of a rectal cancer patient with MSS/PD-L1-negative recurrent hepatopulmonary metastasis who achieved complete remission after treatment with PD-1 inhibitor

**DOI:** 10.3389/fimmu.2023.1197543

**Published:** 2023-07-13

**Authors:** Yang Song, Juan Long, Xiaona Su, Zhuo Chen, Yue He, Weikang Shao, Bin Wang, Chuan Chen

**Affiliations:** ^1^ Department of Oncology, Chongqing Hospital of Traditional Chinese Medicine, Chongqing, China; ^2^ Department of Oncology, Daping Hospital, Army Medical University, Chongqing, China; ^3^ Chongqing Clinical Research Center for Dermatology, Chongqing Key Laboratory of Integrative Dermatology Research, Department of Dermatology, Chongqing Hospital of Traditional Chinese Medicine, Chongqing, China; ^4^ Genecast Biotechnology Co., Ltd, Wuxi, China; ^5^ Department of Oncology, the Seventh People's Hospital of Chongqing (Affiliated Central Hospital of Chongqing University of Technology), Chongqing, China

**Keywords:** MSS, rectal cancer, hepatopulmonary metastasis, immunotherapy, tumor immune microenvironment

## Abstract

Currently, microsatellite high instability (MSI-H)/mismatch repair protein deletion (dMMR) has become a crucial biomarker for utilizing immune checkpoint inhibitors in patients with advanced colorectal cancer (mCRC). However, the proportion of MSI-H/dMMR in advanced patients is only about 5% and mCRC patients with microsatellite stability (MSS)/proficient mismatch repair (pMMR) exhibit poor responses to immunotherapy. Although diverse immune combination therapy regimens have been examined in patients with advanced colorectal cancer who demonstrate MSS/pMMR, these approaches have not yielded favorable efficacy and only a limited proportion of patients have benefited, especially for advanced colorectal cancer patients with liver metastases. Therefore, the mechanism of benefit and potential biomarkers of immunotherapy in patients with MSS/pMMR mCRC deserve more in-depth exploration. Here, we present a case study of a rectal cancer patient with MSS and PD-L1-negative recurrent hepatopulmonary metastases who attained complete remission (CR) and sustained benefits with immunotherapy after systemic therapy had failed. The analysis of the patient’s genetic and immune microenvironmental characteristics revealed that mutations in DNA damage repair (DDR) pathway genes and the existence of abundant tumor-infiltrating lymphocytes could contribute to his potential benefit.

## Introduction

In recent years, immune checkpoint inhibitors have been gradually used in the treatment of mCRC patients and have demonstrated good efficacy, particularly those represented by the anti-PD-1/PD-L1 pathway in mCRC with high microsatellite instability (MSI-H)/mismatch repair protein deficiency (dMMR) (1). However, the proportion of MSI-H/dMMR in advanced patients is only approximately 5%, and most patients with microsatellite stable (MSS) type present limited responsiveness to single immune checkpoint inhibitors. Patients with MSS-type mCRC have a predominantly immune microenvironment characterized by the immune exemption and immune desert type, with low levels of tumor lymphocyte infiltration and tumor mutational load (TMB), and are considered typical “cold tumors” (2). Currently, “combination strategies” and “screening strategies” are mainly employed to improve the efficacy of existing immunotherapy. For example, by combining immunotherapy with radiotherapy, targeted and other local therapies to modulate the immune microenvironment of MSS-type colorectal cancer and transform “cold tumors” into “hot tumors”, or by identifying potential molecular biomarkers for immunotherapy benefit in MSS colorectal cancer.

Studies revealed that vascular endothelial growth factor (VEGF)/vascular endothelial growth factor receptor (VEGFR) inhibitors may synergize with immunotherapy by reducing tumor angiogenesis, promoting vascular normalization, increasing oxygen delivery, and facilitating the infiltration of effector immune T cells (3). Notably, the results of several clinical studies exploring the use of immune combined with anti-angiogenic agents as third-line treatment regimens for mCRC have shown an overall objective remission rate (ORR) of approximately 7%-27%, as well as a median overall survival (OS) of 7.5-15.5 months. These outcomes exhibit a numerical improvement in both ORR and OS compared to the results of previous studies of standard third-line monotherapy (regorafenib, furoquinitinib, or TAS 102). Nevertheless, despite these advancements, the efficacy of this combination remains poor in mCRC patients with liver metastases (4).

In terms of biomarker exploration, there are no accepted predictive biomarkers for the efficacy of immunotherapy for MSS-type colorectal cancer. Previous studies suggested that POLE/POLD1 mutation is an efficacious predictive marker for immunotherapy in solid tumors (5). In addition, a high TMB status is associated with benefit of immunotherapy in MSS-type colorectal cancer, but the clinical significance of TMB remains highly controversial due to limited sample size, different methods of TMB analysis, and high TMB boundaries (6, 7). Furthermore, studies pointed out that tumor infiltrating lymphocytes (TILs), especially CD8+ T cell density, are closely related to the prognosis of colorectal cancer, and the prognostic predictive value of immune score based on the proportion of tumor immune cells is even higher than TNM stage and microsatellite instability status in early to mid-stage colorectal cancer, but the predictive value of immunotherapy efficacy in advanced intestinal cancer is unclear (8). Therefore, it is imperative to identify additional biomarkers to guide the screening of individuals who can benefit from immunotherapy for MSS-type colorectal cancer.

The present study reports a patient with MSS/PD-L1-negative recurrent hepatopulmonary metastatic rectal cancer who achieved complete remission (CR) using immunotherapy after systemic therapy failure, with PFS over 41 months and OS over 106 months. To gain insight into the potential benefit mechanism, the genome and tumor immune microenvironment were characterized using next-generation sequencing (NGS) and multiplex immunohistochemistry (mIHC).

## Case presentation

In January 2019, a 50-year-old male patient with liver metastasis from rectal cancer was admitted to the hospital. The CT findings showed a major metastasis (7.1×4.9 cm) in the left lobe of the right liver and caudate lobe. A puncture pathology biopsy of the liver metastasis (Tumor3) indicated metastatic rectal adenocarcinoma in the right liver. The results of next generation sequencing (NGS) revealed KRAS wild type, MSS and PD-L1 negative, and TMB-H (11.7 Muts/Mb). The patient received 8 cycles of conversion therapy (4 cycles each of FOLFIRI+Bevacizumab and FOLFOX6+Bevacizumab) at the initial diagnosis of stage IV rectal adenocarcinoma with left liver metastasis in March 2014. Subsequently, radical surgery was performed for rectal and liver metastatic tumors in October and November 2014, respectively. The NGS results of colonoscopic biopsy tissue (Tumor1) at initial diagnosis suggested KRAS p.G12D mutation, while the NGS results of rectal surgery tissue (Tumor2) in October 2014 indicated KRAS wild type, MSS, and TMB-L (1.9471 Muts/Mb). After surgery, the patient received 5 cycles of adjuvant chemotherapy (CapeOX+Bevacizumab) and 1 year of maintenance therapy (Capecitabine+Bevazizumab) until disease progression in January 2019.

After admission, the patient received 3 cycles of FOLFIRI combined with Cetuximab conversion therapy and underwent right hepatic tumor resection in March 2019. The NGS results of surgical tissue (Tumor4) from right liver metastases suggested KRAS wild type, MSS, PD-L1 negative, and TMB-M (3.81 Muts/Mb). A repeat MRI conducted one month after surgery showed multiple abnormal nodal shadows in the liver. Subsequently, the patient was treated with CapeOX combined with the Bevacizumab regimen, and due to intolerable adverse effects during oxaliplatin, the treatment regimen was adjusted to XELIRI combined with Bevacizumab. However, in August, chest CT and abdominal MRI revealed multiple metastases in both lungs and liver and disease progression.

**Figure 1:**
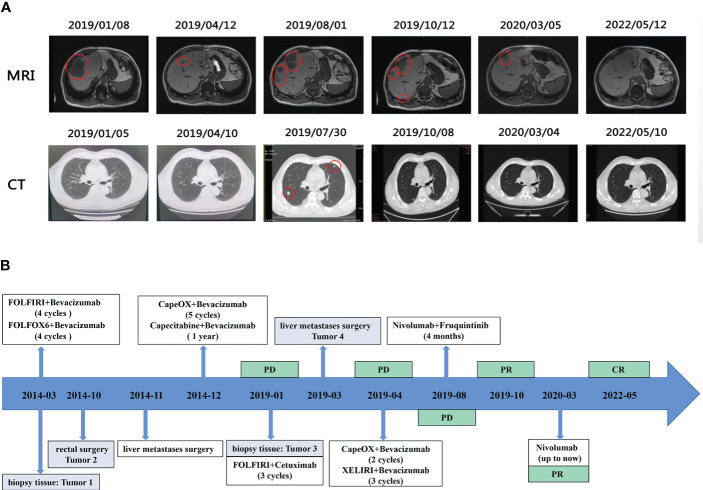
**(A)** Representative imaging images of the treatment process. **(B)** Timeline of the patient’s treatment process.

With the consent of the patient and his family, a decision was made to try nabritumomab in combination with furoquinitinib in August 2019. Surprisingly, 2 months later, the chest CT showed that both lung tumors had almost disappeared, and an abdominal enhanced MRI indicated a significant reduction in liver lesions compared to the previous one. Moreover, a review in March 2020 revealed a continued reduction of liver lesions and an efficacy evaluation of PR. Subsequent maintenance treatment with only nivolumab monotherapy has been administered to date, with the complete disappearance of liver and lung tumors to CR at the last review in May 2022. Currently, the patient is in good physical condition with a PFS of 41 months and an OS of 106 months of OS. The imaging and treatment course of this patient are detailed in **Figure 1**.

## Gene analysis

**Figure 2:**
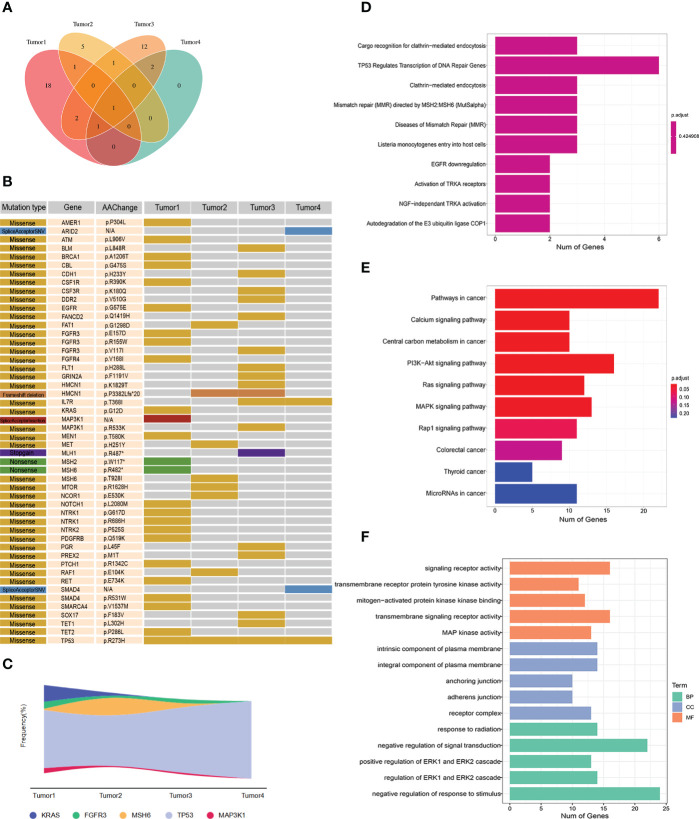
**(A)** Wayne diagram showing the distribution of somatic mutations in four tumor tissues. **(B)** Mutation profiles of four tumor tissues. **(C)** Mutation frequency of some genes in four tumors. **(D)** Reactome analysis of tumor mutations. **(E)** KEGG analysis of tumor mutations. **(F)** GO analysis of tumor mutations.

Two formalin-fixed paraffin-embedded (FFPE) tumor tissue samples (Tumor2 and Tumor3) were sent again for liquid-phase hybridization capture second-generation sequencing (Genecast Biotechnology Co., Ltd, Wuxi). The tissue DNA was extracted with the blackPREP FFPE DNA Kit (Analytik Jena, Germany). Genomic DNA were shewered into 150- to 200-bp fragments with a Covaris M220 Focused-Ultrasonicator (Covaris, Massachusetts, USA). A designed panel of the IDT library(Integrated DNA Technologies, USA) was used to capture DNA libraries. The captured samples were sequenced using a panel consisting of 769 genes. The Venn diagram illustrated the distribution of somatic mutations in four different tumor tissues (**Figure 2A**). The gene mutation mapping revealed the KRAS G12D mutation in Tumor1, but after treatment, no KRAS mutation was found in Tumor2, and the remaining two liver metastases were also KRAS wild type. It was observed that colorectal cancer patients whose KRAS changed from mutant to wild type had a better survival prognosis compared to those with persistent KRAS mutation, and patients with KRAS wild-type colorectal cancer liver metastases had a better prognosis (9, 10). Additionally, we found that Tumor2 and Tumor4 had fewer tumor mutation types compared to Tumor1 and Tumor3, respectively, suggesting the suppression of the molecular evolution of the tumor during treatment (**Figures 2B, C**). Moreover, we observed the molecular mutational load reduced in the course of treatment. However, TP53 p.R273H persisted and the mutation frequency was increased in Tumor3 and Tumor4, which could be associated with the development of liver metastases in patients. The Reactome enrichment analysis showed that the patient’s mutations were primarily enriched in the DDR pathway, indicating the sensitivity of the patient to immunotherapy (11). The KEGG and GO analysis demonstrated the enrichments of genes were mainly in PI3K-Akt signaling pathway and negative regulation of response to stimulus, which may explain the tumor proliferation and metastasis in the ealry stage of treatment (**Figures 2D-F**).

## Multiple immunohistochemical test

**Figure 3:**
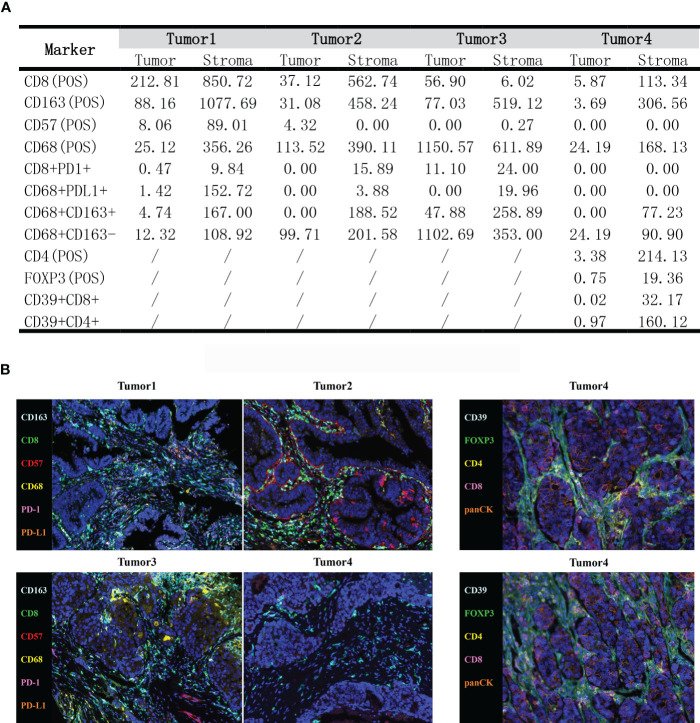
**(A)** The density of tumor-infiltrating immune cells in the tumor immune microenvironment by mIHC (cells/mm^2^). **(B)** Typical images showing the expression of all markers in different tumor tissues.

Multiple immunohistochemistry was performed to investigate the patient’s immune microenvironment with the panel consisting of CD8, CD68, CD163, CD57, PD-L1 and PD-1. Another panel including CD8, CD4, CD39, FOXP3 and panCK was detected in tumor4 to further explore the microenvironment. The 4-μm serial tumor sections were stained using the Opal 7-Color fIHC Kit (Akoya Biosciences, MA) based on the Tyramide Signal Amplification (TSA). The slides were subjected to epitope retrieval in Tris-EDTA buffer (pH 9.0; Zsbio, Beijing, CN) after deparaffinage and rehydration. Endogenous peroxidases were blocked by incubating in Antibody Diluent/Block (Akoya Biosciences, MA). The stained slides were scanned using the TissueFACS SL plus S system (TissueGnostics, Vienna, Austria; acquisition software: TissueFAXS SL V7.1.120) and analyzed by Advanced Image Analysis software StrataQuest (V7.1.1.129). The results showed that the immune microenvironment of this patient was enriched in immune cells, especially CD8^+^ T cells, CD68^+^ macrophages, and CD163^+^ macrophages, with a relatively high percentage of M1-type macrophages (CD68^+^CD163^-^). In addition, CD4^+^ T cells and a few CD39^+^CD4^+^, CD39^+^CD8^+^ were infiltrated in Tumor4, which implied the favorable response to immunotherapy. Interestingly, we also found FOXP3^+^ T cells infiltration in the TME of Tumor4, despite the common view that FOXP3^+^ T cells were immunosuppressive, there were studies indicated FOXP3^+^ T cells were associated with good prognosis of CRC (12–14) (**Figure 3**).

## Discussion

The current 3-year survival rate for mCRC patients is only about 30%, and the survival rate is even lower for MSS/pMMR patients. The efficacy of immunotherapy in MSS/pMMR mCRC patients is limited, particularly in MSS/pMMR mCRC patients with liver metastases, and it was shown that the ORR of immunotherapy is only 1.9% and the PFS is 5.5 (IQR, 2.0-11.5) months (15–17). In a recent retrospective study, the effectiveness of furoquinitinib + PD-1 inhibitor was compared to regorafenib + PD-1 inhibitor in patients with refractory MSS mCRC. The results revealed that the DCR rate and PFS were prominently higher with furoquinitinib plus PD-1 inhibitor than with regorafenib plus PD-1 inhibitor, but there was no statistically significant difference between the two groups in patients with liver metastases. Nevertheless, in this case, we decided to try furoquinitinib plus PD-1 monoclonal antibody, and to our surprise, the tumor continued to shrink and eventually reached CR with the subsequent use of PD-1 monoclonal antibody alone.

In this case, the patient was initially diagnosed with a KRAS mutation in the biopsy tissue and all other tissue specimens following treatment showed indications of KRAS wild type. Studies demonstratedthat patients with colorectal cancer who undergo a transformation from mutant to wild-type KRAS have a more favorable survival prognosis (9, 10). In CRC, RAS mutant and RAS wild-type cells always coexist in the same tumor microenvironment in a balanced manner, competing for space and resources (18). Tumors with RAS mutation are under negative selection pressure from chemotherapy or antiangiogenic therapy to support the growth of RAS wild type clones, resulting in their relative increase (19). Meanwhile, the cancer stem cell (CSC) model showed that tumor cells with stem cell characteristics could self-renew indefinitely and might achieve multi-directional differentiation between RAS mutation and wild type (20). Therefore, the heterogeneity of subclones of the tumor itself, the presence of CSC and the effects of chemotherapy or antiangiogenic therapy may contribute to the occurrence of RAS mutation conversion. TP53 p.R273H is the only shared variant among the four lesions and has an elevated mutation abundance in liver metastases. TP53 is known to be an oncogene, and its mutation not only impairs the role of wild-type p53 in inhibiting tumor proliferation but also confers an oncogenic function to the gene. In addition, research confirmed that TP53 mutations are associated with chemoresistance in colorectal cancer patients, which may contribute to the recurrence of liver metastases and poor efficacy of subsequent chemotherapy in this patient (21). Overall, the mutation-enriched pathway of this patient was mainly the DDR pathway, and several studies suggested that alterations in DDR pathway genes can be used as a potential biomarker to predict the benefit of immunotherapy in patients with advanced colorectal cancer. When the repair function of the DDR pathway is disrupted, the frequency of genomic mutations in tumor cells increases, and abnormal proteins produced by the mutated genes escalate accordingly, rendering it more probable that neoantigens will be produced to activate the immune system. In other cancer types such as breast, ovarian, and pancreatic cancers, DDR mutations have also been reported to be associated with an increase in tumor-infiltrating lymphocytes and an immunogenic gene signature within the tumor (22, 23). Therefore, DDR gene mutations in patients with advanced colorectal cancer may expand the range of populations that can benefit from immunotherapy, especially when screening MSS-type mCRCs for potential ICI benefits, which have far-reaching clinical implications.

Previous research illuminated that a small proportion of patients with pMMR/MSS mCRC can still benefit from anti-PD-1/PD-L1 immunotherapy (24). The tumor microenvironment (TME) provides additional reference information, and TILs are an important component of the TME, affecting tumor growth, metastasis, and the efficacy of immunotherapy. CD8^+^ T cells, a major driver of anti-tumor immunity, mediates tumor rejection by recognizing tumor antigens and directly kill tumor cells. Moreover, a study showed that the expression of PD-1 on CD8^+^ T cells was also associated with improved survival of CRC patients, especially for MSS CRC (25). Meanwhile, CD4^+^ T cells could promote the recruitment and activation of CD8^+^ T cells, macrophages and natural killer (CD57^+^) cells to enhance the antitumor effect. Studies revealed CD39 could serve as a marker for identification of tumor-specific T cells and CD39^+^CD8^+^, CD39^+^CD4^+^TILs were found to be beneficial for antitumor activity (26, 27). Interestingly, FOXP3^+^ T cells are usually regarded as suppressive T cells in many cancers except CRC, in which FOXP3^+^ T cells indicated better prognosis in some studies (12–14). In fact, study has shown FOXP3^+^ T cells are functionally and phenotypically heterogeneous and include suppressive and non-suppressive subpopulations (12). In our case, we speculated FOXP3^+^ T cells might mainly play a nonsuppressive role in the TME and generate a favorable microenvironment. Tumor-associated macrophages (TAMs, CD68^+^ macrophages), one of the most abundant immune cells in the TME, were generally related to better survival despite conflicting findings in colorectal cancer (28). Besides, the PD-L1 expression on CD68^+^ macrophages were reported to be associated with improved outcome. Macrophages are usually classified into two phenotypes: classically activated macrophages (M1, CD68^+^CD163^-^) and selectively activated macrophages (M2, CD68^+^CD163^+^). M1-type macrophages promote antitumor immune responses by regulating antigen presentation and secreting proinflammatory cytokines, whereas M2-type macrophages exert immunosuppressive effects. However, it has also been found that M2 macrophages in the TME were favorable for the prognosis of CRC patients (29, 30). In the present case, the presence of both macrophages seemed to favor immunotherapy. In conclusion, these abundant immune cells in the patient’s TME exerted an important role in the immunotherapy.

In conclusion, we report a case of a patient with MSS, PD-L1-negative recurrent hepatopulmonary metastatic rectal cancer who achieved CR after treatment with an immune checkpoint inhibitor, with an overall PFS of 41 months and an OS of more than 106 months, and is continuing to benefit. This case demonstrates that immunotherapy can also provide good survival benefit for patients with MSS-type advanced colorectal cancer, and the underlying mechanism and new effective predictive markers for immunotherapy in MSS-type colorectal cancer patients deserve more in-depth investigations in the future.

## Data availability statement

The original contributions presented in the study are included in the article/**Supplementary Material**. Further inquiries can be directed to the corresponding authors.

## Ethics statement

The studies involving human participants were reviewed and approved by Ethics Committee of Army Medical Center of PLA Approval of Medical Research Involving People Ethical. The patients/participants provided their written informed consent to participate in this study. Written informed consent was obtained from the participant/patient(s) for the publication of this case report.

## Author contributions

YS and JL supported the article writing and the clinical data collation of clinical data. YH was mainly responsible for writing and performed the gene and mIHC analysis. WS was in charge of figure-making. CC and BW were the corresponding authors. All authors contributed to the article and approved the submitted version.
